# Editorial: Galectins and hormones in health and disease

**DOI:** 10.3389/fendo.2025.1545421

**Published:** 2025-02-05

**Authors:** María F. Troncoso, Roger Chammas, Vinícius Frias Carvalho, Felipe Leite Oliveira, Dea Maria Serra Villa-Verde

**Affiliations:** ^1^ Departamento de Química Biológica, Facultad de Farmacia y Bioquímica, Universidad de Buenos Aires, Buenos Aires, Argentina; ^2^ Instituto de Química y Fisicoquímica Biológicas (IQUIFIB) Prof. Alejandro C. Paladini, Consejo Nacional de Investigaciones Científicas y Técnicas (CONICET)-Universidad de Buenos Aires, Buenos Aires, Argentina; ^3^ Center for Translational Research in Oncology, Instituto do Câncer do Estado de São Paulo, Hospital das Clínicas da Faculdade de Medicina da Universidade de São Paulo, São Paulo, Brazil; ^4^ Comprehensive Center for Precision Oncology, Universidade de São Paulo, São Paulo, Brazil; ^5^ Laboratory of Inflammation, Oswaldo Cruz Institute, Oswaldo Cruz Foundation, Rio de Janeiro, Brazil; ^6^ Laboratório de Interações Celulares, Instituto de Ciências Biomédicas, Federal University of Rio de Janeiro, Rio de Janeiro, Brazil; ^7^ Laboratory on Thymus Research, Oswaldo Cruz Institute, Oswaldo Cruz Foundation, Rio de Janeiro, Brazil

**Keywords:** galectin, immune system, homeostasis, diabetes, ageing, cancer

Galectins are a family of β-galactoside-binding lectins with essential roles in various biological processes, such as tissue repair, adipogenesis, immune cell homeostasis, angiogenesis, and pathogen recognition (1, 2). Notably, altered expression of galectins or disruptions in their interactions with glycan partners are associated with a wide range of pathological conditions, including cancer, autoimmune inflammation, infections, fibrosis, and metabolic disorders (3, 4). Particularly, research on galectin-3 (Gal-3), a member of the galectin family, primarily targets its inhibition due to its role in promoting cancer and metastasis, presenting the potential for anticancer therapy (5, 6). Recent evidence also indicates that Gal-3 is overexpressed in various metabolic disorders, including diabetes, obesity, and atherosclerosis, and plays a role in regulating the onset and progression of these conditions (7). Studies in clinical research have shown a strong association between high levels of circulating Gal-3 and the occurrence of diabetes and its related complications (8). There is substantial evidence that Gal-3 plays a role in the development of diabetic complications by acting as a receptor for advanced glycation end-products and advanced lipoxidation end-products (9). Furthermore, increased expression of Gal-3 in pancreatic β-cells modulates glucose metabolism and glycoregulation in mice on a high-fat diet, impacting both fasting glucose levels and overall glycemia (10). Collectively, this evidence underscores the importance of understanding how galectins modulate hormonal responses and their potential impact on developing new therapies.

The current Frontiers in Endocrinology Research Topic, entitled *Galectins and Hormones in Health and Disease*, explores the complexities of the galectin-hormone association and how its modulation can influence human physiology and pathology. In this context, the article by Souza et al. highlights the multifaceted role of Gal-3 as a hormonal regulator and its potential as a therapeutic target in hormone-resistant cancers. Souza et al. examine the role of Gal-3 in prostate cancer development and progression, particularly focusing on its interaction with estrogen receptors (ER) in castration-resistant prostate cancer (CRPC). Gal-3 is frequently dysregulated in various cancers, and in prostate cancer, its interaction with estrogen receptors (ERα and ERβ) appears to influence tumor cell proliferation, migration, and invasion, particularly in androgen-independent cells. The authors propose that Gal-3 and ER may work in concert to modulate nuclear transcription mechanisms, affecting genes linked to tumor growth and apoptosis resistance. Additionally, the article discusses how Gal-3 can promote a pro-tumor environment by diminishing immune responses against the tumor. This focus on the hormonal regulation of Gal-3 in hormone-dependent cancers offers promising insights for new therapeutic targets, as understanding this interaction is essential for developing targeted treatments for CRPC. Thus, this study provides a unique perspective on Gal-3’s role in hormonal signaling in prostate cancer and its contribution to hormonal treatment resistance—a significant challenge in CRPC.

In addition, studies in mice have shown that Gal-3 deficiency disrupts thymus homeostasis, leading to increased local and systemic glucocorticoid levels and signs of premature thymic involution (11). The article by Ramos et al. focuses on Gal-3 distribution and its role in the thymus of pre-diabetic non-obese diabetic (NOD) mice. It highlights how Gal-3 exhibits altered expression in the NOD mouse thymus. This change impacts thymocyte migration, with Gal-3 found in association with specific thymic cells and extracellular matrix molecules. Notably, Gal-3 clustering with B lymphocytes and dendritic cells within the thymic perivascular spaces (PVS) suggests a potential role in immune modulation linked to autoimmune diabetes. Findings reveal that NOD thymocytes exhibit impaired migration in response to Gal-3, which may contribute to the autoimmune processes in type 1 diabetes.

Gal-3 and Matrix Metalloproteinase-9 (MMP-9) have been associated with the pathophysiology of atherosclerosis. Both proteins are involved in inflammatory processes, plaque instability, and tissue remodeling (12, 13). The mutual contribution of these proteins has made them potential biomarkers for assessing the severity and risk of cardiovascular events in patients with atherosclerosis. In this context, Liu et al. used *in vitro* and *in vivo* strategies to investigate a possible association between Gal-3 and MMP-9 as early markers of atherosclerosis in diabetic patients. *In vitro* data revealed that active human MMP-9 increased the gene and protein expression of MCP-1, ICAM-1, and VCAM-1 in human coronary artery smooth muscle cells (HCASMCs).They also demonstrated that exogenous MMP-9 induced both inflammation and atherosclerosis in diabetic KK.Cg-Ay/J (KK) mice, with significant correlation with macrophages expressing Gal-3 in the carotid arteries. In diabetic patients, the serum levels of MMP-9 were linked to size and number of carotid artery plaques, and luminal stenosis of coronary arteries.

Furthermore, clinical data suggest that plasma levels of Gal-3 are associated with heart failure risk (14). While the exact roles of Gal-3 in the pathophysiology of cardiovascular diseases are still unclear, clinical research supports measuring its levels in certain patient cohorts. The profibrotic activity of Gal-3 (3, 15) and its involvement in atherosclerosis (16) contribute to the clinical relevance of this lectin, spurring investigations into the clinical settings and the potential use of Gal-3 inhibitors (17, 18). Nevertheless, studies that shed light on the clinical significance of Gal-3 are still necessary. Cao et al. present a phase IV clinical protocol in which they will follow two cohorts of diabetic patients, an experimental group receiving sodium/glucose transporter inhibitors (iSGLT2) and a control group receiving conventional treatment for type II diabetes. Among the variables followed, the authors will study plasma levels of Gal-3, correlating them with cardiac function in diabetic patients treated or not with iSCGLT2. Similar studies will be necessary to identify clinical conditions that may benefit from Gal-3 targeted therapeutic strategies.

In conclusion, galectins, particularly Gal-3, play critical roles at the intersection of Endocrinology and various pathological conditions, including cancer, diabetes, and cardiovascular diseases. Current research underscores the significance of these glycan-binding proteins in modulating hormonal responses and their implications for key biological processes such as tumor growth and immune regulation. As we gain a deeper insight into the association between galectins and hormones, new therapeutic strategies may emerge that could improve clinical outcomes in challenging diseases. Ongoing research into this association will be crucial for furthering personalized medicine approaches in the management of metabolic and cancer-related conditions.
